# High-performance and flexible thermoelectric films by screen printing solution-processed nanoplate crystals

**DOI:** 10.1038/srep33135

**Published:** 2016-09-12

**Authors:** Tony Varghese, Courtney Hollar, Joseph Richardson, Nicholas Kempf, Chao Han, Pasindu Gamarachchi, David Estrada, Rutvik J. Mehta, Yanliang Zhang

**Affiliations:** 1 Micron School of Materials Science and Engineering, Boise State University, Boise, ID 83725, United States; 2 Department of Mechanical and Biomedical Engineering, Boise State University, Boise, ID 83725, United States; 3 Department of Electrical and Computer Engineering, Boise State University, Boise, ID 83725, United States; 4 ThermoAura Inc. 132 B. Railroad Avenue, Colonie, NY 12205, United States

## Abstract

Screen printing allows for direct conversion of thermoelectric nanocrystals into flexible energy harvesters and coolers. However, obtaining flexible thermoelectric materials with high figure of merit ZT through printing is an exacting challenge due to the difficulties to synthesize high-performance thermoelectric inks and the poor density and electrical conductivity of the printed films. Here, we demonstrate high-performance flexible films and devices by screen printing bismuth telluride based nanocrystal inks synthesized using a microwave-stimulated wet-chemical method. Thermoelectric films of several tens of microns thickness were screen printed onto a flexible polyimide substrate followed by cold compaction and sintering. The n-type films demonstrate a peak ZT of 0.43 along with superior flexibility, which is among the highest reported ZT values in flexible thermoelectric materials. A flexible thermoelectric device fabricated using the printed films produces a high power density of 4.1 mW/cm^2^ with 60 °C temperature difference between the hot side and cold side. The highly scalable and low cost process to fabricate flexible thermoelectric materials and devices demonstrated here opens up many opportunities to transform thermoelectric energy harvesting and cooling applications.

Thermoelectric conversion is a solid-state and environmentally friendly energy conversion technology with broad applications including solid-state cooling, energy harvesting, and waste heat recovery^1^. Flexible thermoelectric devices are especially attractive for waste heat recovery along contoured surfaces and for energy harvesting applications to power sensors, biomedical devices, and wearable electronics – an area under exponential growth^2^.

The efficiency of thermoelectric materials is determined by the figure of merit ZT defined as ZT = α^2^σ*T*/κ, where α, σ, κ and *T* are the Seebeck coefficient, electrical conductivity, thermal conductivity, and absolute temperature respectively^3^,^4^. Nanostructured thermoelectric materials have been widely studied in recent years and proven to have unique and superior thermoelectric performance compared to their bulk counterparts due to the ability to tailor electron and phonon transport and effectively increase ZT^5^,^6^,^7^. Despite significant ZT improvements in nanostructured materials^8^,^9^,^10^, the lack of scalable and low-cost manufacturing processes remains a major challenge to the wide use of these materials^11^. In addition, major progress in ZT enhancement through nanostructuring has historically been achieved in mechanically rigid materials, while flexible thermoelectric materials are still relatively unexplored and have fairly low ZT^2^.

Among all the methods to fabricate thermoelectric materials, wet deposition of nanocrystal-based colloidal inks using screen printing, inkjet printing, direct writing, or other layer-by-layer methods hold many advantages due to the ability to directly convert nanocrystal inks into micro/macroscale functional materials and devices with great scalability, flexibility, and cost effectiveness^12^,^13^. Using inkjet or disperser printing, several research groups have achieved ZT of ~0.3 in thermoelectric films printed on flexible substrates^14^,^15^. Screen printing has also been explored as a more efficient way to fabricate thermoelectric devices^16^,^17^,^18^,^19^. Despite the above proof-of-concept demonstrations, flexible thermoelectric films fabricated by printing methods continue to exhibit fairly low ZT in the 0.1–0.3 range, significantly lower than their rigid bulk counterparts fabricated using conventional approaches such as hot press or spark plasma sintering^2^. There are many challenges in printing efficient and flexible thermoelectric materials using nanocrystals, including scalable synthesis of high-performance nanocrystals, nanocrystal surface oxidation during printing processes, and poor density and electrical conductivity of the printed films^20^.

Here, we report a study of flexible thermoelectric films by screen printing colloidal inks composed of bismuth telluride based nanoplates fabricated using a scalable microwave-stimulated wet chemical approach^21^ (shown in Fig. 1). The peak ZT of our flexible films reaches 0.43 at 175 °C due to a combination of high power factor and low thermal conductivity, which is among the highest ZT reported for flexible thermoelectric materials fabricated by printing. The films demonstrate superior flexibility with negligible changes in electric resistance with 150 bending cycles. In addition to the unprecedented high ZT and flexibility, another significant advantage of our work is the use of thioglycolic acid (TGA) as a surface capping agent to inhibit nanocrystal oxidation^21^, thus enabling large-scale manufacturing at ambient conditions.

## Methods

### Nanocrystal ink synthesis

Our doped and functionalized pnictogen chalcogenide nanocrystals of Bi_2_Te_2.8_Se_0.2_ were synthesized using a microwave stimulated wet-chemical synthesis method based on inexpensive organic solvents and metal salts described earlier^21^. In this method, the reaction between molecularly ligated chalcogen and pnictogen complexes was activated by microwave stimulation with the presence of thioglycolic acid (TGA), which serves as a shape-directing, oxide-inhibiting and sulfur-dopant delivery agent. The resulting precipitate is cleaned and dried in ambient conditions to obtain powders consisting of single-crystal nanoplates of 5- to 20-nm-thickness with bounding edge dimensions ranging between 50 to 500 nm.

The dried nanocrystals with the TGA capping agent were mixed with solvent and binder to produce viscous and thixotropic inks for screen printing. The optimized ink contains 58 wt.% Bi_2_Se_2.8_Se_0.2_ nanopowders, 39 wt.% Solvent (α-Terpineol, from Sigma-Aldrich), 2 wt.% Binder (Disperbyk-110, from BYK U.S.A. Inc.), 1 wt.% Glass Frits (from Artglass Supplies, 325 mesh). The ink is thoroughly mixed using a planetary centrifugal mixer for 20 minutes followed by a vortex mixer for 10 minutes to get a uniform mixture.

### Flexible film fabrication

As-prepared ink is screen printed on flexible polyimide substrates. The thermoelectric films of various thicknesses in the range of 10–100 μm were obtained by controlling the screen mesh size and the number of repeated print passes. The printed films were first dried in air at 200 °C on a hot plate to remove the solvent and binder, followed by a cold compaction using a hydraulic press to consolidate the films. The film was finally sintered at 430 °C for 45 minutes in vacuum in order to remove the TGA surfactant and further improve the film density. The sintering temperatures are kept below the melting point of the polyimide substrate, though better thermoelectric properties could be obtained at higher sintering temperatures.

### Thermoelectric property measurement

The temperature-dependent in-plane electrical conductivity and Seebeck coefficient of the film sample were measured simultaneously using a commercial Linseis Seebeck and resistivity instrument. The above two properties of the same sample were also measured using a home-built testing system, and the two sets of measurement results are within 2%. In order to measure the thermal conductivity of the sample, a freestanding film of about 100 μm thickness was prepared under the same conditions as those for preparing thinner films on substrate. The temperature-dependent cross-plane thermal diffusivity of the freestanding film was measured using a laser flash instrument. The cross-plane thermal conductivity was then determined using the sample density measured using Archimedes method and the specific heat measured using a DSC instrument. The in-plane thermal conductivity of the freestanding film was measured directly using a steady-state method in vacuum, which is within 5% of the cross-plane thermal conductivity, indicating the sample is isotropic. Details about the thermal conductivity measurement are included in the supplementary information. The carrier concentration and mobility were measured using Hall measurement conducted on the Physical Property Measurement System (PPMS) with 4 wire connection and the magnetic field sweeping from −1 T to 1 T.

### Thermoelectric device fabrication and testing

Five 10 mm × 2 mm × 0.01 mm n-type Bi_2_Te_2.8_Se_0.2_ elements were printed onto a flexible polyimide substrate with 4 mm spacing. Thin copper foils were soldered to the five elements in order to connect them electrically in series. A custom test bed was built using two commercial Peltier modules, one operating as a heater and the other as a cooler. The hot side and cold side of the TE device were thermally grounded to the two Peltier modules to create a temperature gradient. Two 75 *μ*m diameter k-type thermocouples were mounted to the hot and cold sides of the device to measure the temperature difference. The device was connected electrically in series with a shunt resistor and a variable resistor for impedance matching at each measurement temperature. The open circuit voltage, load voltage, current, internal resistance, and power output from the device were measured at each hot-side temperature while the cold-side temperature is maintained constant.

## Results and discussion

**Figure 2:**
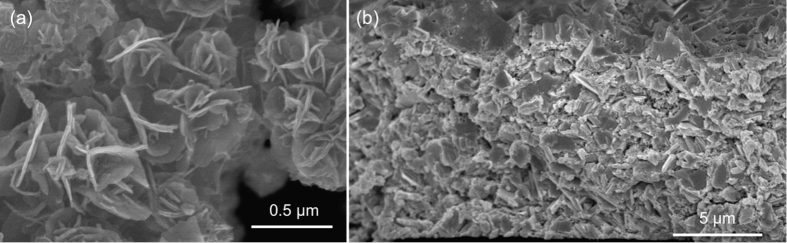
SEM images of (**a**) the Bi_2_Te_2.8_Se_0.2_ nanocrystals and (**b**) the cross section of a printed film on polyimide substrate.

Figure 2(a) shows scanning electron microscope (SEM) images of the Bi_2_Te_2.8_Se_0.2_ nanocrystals, indicating the plate-like structures of several tens of nanometers thickness. Figure 2(b) shows a cross-section SEM image of a flexible Bi_2_Te_2.8_Se_0.2_ film of about 10 μm thickness fabricated by screen printing. The films have about 85% relative density, and contain nanoscale pores primarily due to incomplete sintering of the nanocrystals and the removal of the additives in the ink.

**Figure 3:**
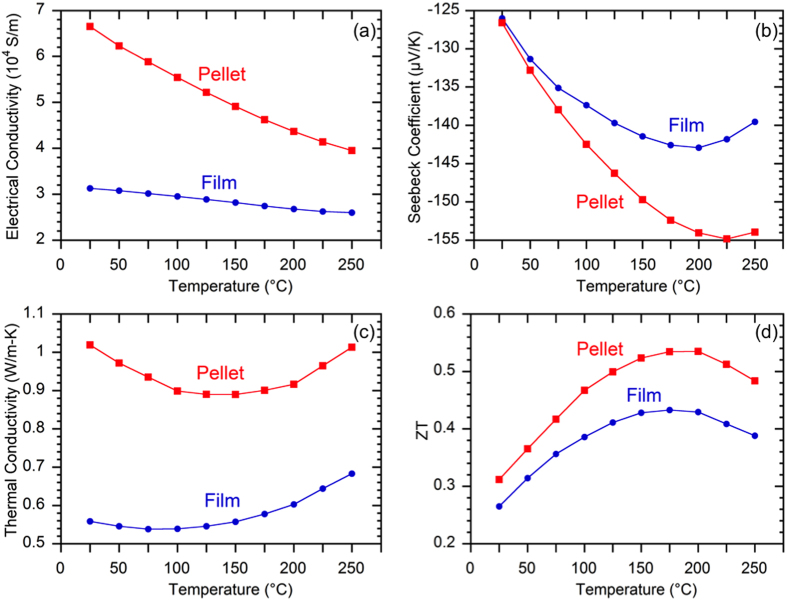
Temperature-dependent (**a**) Electrical conductivity (**b**) Seebeck coefficient (**c**) Thermal conductivity and (**d**) ZT of a 10 μm thick flexible film fabricated by printing the nanoplate ink and a 500 μm thick reference pellet fabricated by cold-compaction and sintering of the pure nanoplate powders.

Temperature-dependent thermoelectric properties were obtained on a flexible film of 10 μm thickness printed using the nanocrystal ink and a reference pellet sample of 500 μm thickness made by the pure nanocrystal powders using cold compaction and sintering under the same conditions. The relative densities of the film and the pellet are 85% and 90% respectively. As shown in Fig. 3(a), the room-temperature electrical conductivity of the film is about 53% lower than the pellet. Figure 3(b) shows the Seebeck coefficients of the two samples are within 10% for the entire measurement temperature, indicating approximately the same carrier concentrations for both the film and the pellet. Indeed, the Hall measurement validated that the carrier concentrations of the film and the pellet are within 10% (1.56 × 10^19^ cm^−3^ versus 1.42 × 10^19^  cm^−3^), whereas the film mobility is about 56% lower than the pellet mobility (127 cm^2^V^−1^s^−1^ versus 290 cm^2^V^−1^s^−1^) due to increased electron scattering by impurities and porosity present in the printed films.

The room-temperature lattice thermal conductivity κ_L_ of the film and the pellet is estimated to be 0.41 Wm^−1^K^−1^ and 0.66 Wm^−1^K^−1^, respectively, using the equation κ_L_ = κ − σTL, where κ is the total thermal conductivity, σ is the electrical conductivity, T is the absolute temperature, and L is the Lorenz number determined from our previous work^22^. The κ_L_ of these samples is significantly lower than their bulk counterpart attributed to the nanoscale grains and porosities originated from the nanocrystals^22^. Furthermore, the κ_L_ of the film is lower than the pellet mainly due to additional phonon defects scattering caused by the addition of glass particles as well as a small contribution from slightly higher porosities.

As shown in Fig. 3d, the film demonstrates a peak ZT of 0.43 at 175 °C, which is only 20% lower than control pellet despite 53% lower electrical conductivity. The significantly reduced thermal conductivity largely compensates the electrical conductivity losses and contributes to the high ZT in the printed films. In comparison, Table 1 summarizes the peak ZT (or room-temperature ZT if peak ZT is not available) of several representative n-type flexible thermoelectric materials, summarizing the highest reported ZT thus far in each category. The peak ZT of our flexible film is significantly higher than the previously reported bismuth telluride materials fabricated by printing, and is also among the highest reported value in all the reported n-type flexible thermoelectric films.

**Figure 4:**
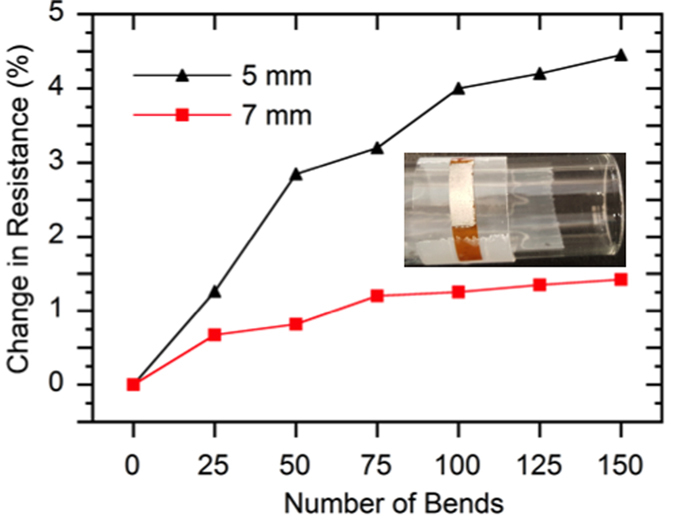
Percentage increase of electrical resistances of flexible films as a function of number of bending cycles for 7 mm bending radius and 5 mm bending radius.

In order to test the flexibility, the room-temperature electrical resistance of the printed films was tested using Van der Pauw method as a function of bending cycles on two cylinders of 7 mm radius and 5 mm radius respectively. Electrical resistance is chosen here to evaluate film flexibility because it is very sensitive to any cracks that may develop during bending test. After 150 bending cycles, the electrical resistances of the film show 1.4% increase for the 7 mm bending radius and 4.5% increase for the 5 mm bending radius respectively, indicating superior bending flexibility (shown in Fig. 4).

**Figure 5:**
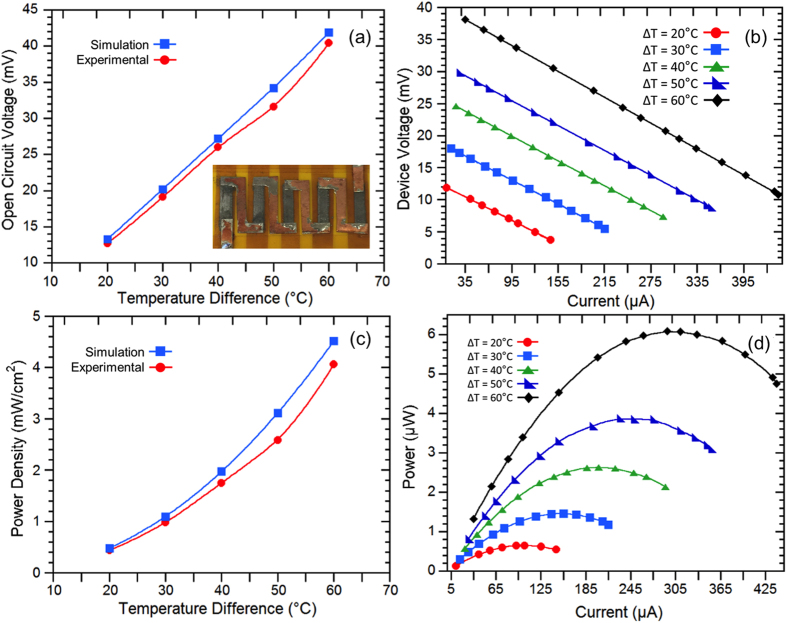
Testing results of a thermoelectric device fabricated by the screen printed flexible films. (**a**) Experimental and calculated open circuit voltage vs. temperature differences (ΔT), (**b**) Device operating voltage vs. current tested at various ΔT, (**c**) Experimental and calculated electrical power density vs. ΔT (**d**) Electrical power output tested at various ΔT. Inset in (**a**) is a picture of the device.

A thermoelectric generator device consisting of 5 n-type elements (shown in Fig. 5(a) inset) was fabricated in order to validate the performance of the flexible films. Figure 5 shows the experimental and simulation results of the device tested at different temperature differences (ΔT) when the hot side temperature was varying from 40–80 °C and the cold side was maintained at 20 °C. The open circuit voltage, device voltage during operation, and power output increase as the ΔT increases. As shown in Fig. 5(a,c), the maximum open circuit voltage and power density reach 41 mV and 4.1 mW/cm^2^ with 60 °C ΔT. The power density was determined based on the total cross-sectional area (2 mm × 0.01 mm × 5) of the five thermoelectric elements. The experimental results are within 10% of the finite element simulation results based on the thermoelectric properties shown in Fig. 3, which further verified the measured film properties. Figure 5(b,d) shows the device operating voltage and power output as a function of electrical current tested by varying the external load resistances. The maximum power of 6.1 μW is obtained with 60 °C ΔT when the external load resistance matches with the internal resistance of the device.

## Conclusions

Flexible thermoelectric films were screen printed at ambient conditions using nanocrystals synthesized by a highly scalable microwave assisted wet chemical method. The films show an unprecedented peak ZT of 0.43 at 175 °C and superior flexibility with negligible changes of electrical conductivity after 150 bending cycles. The flexible thermoelectric device fabricated using the printed n-type thermoelectric elements produces a high power density of 4.1 mW/cm^2^ with a small temperature difference of 60 °C, opening up lots of applications for low-temperature energy harvesting. The performance of the printed thermoelectric films and devices can be further improved by increasing the electrical conductivity through optimization of the ink formulation and refinement of the sintering process.

## Additional Information

**How to cite this article**: Varghese, T. *et al*. High-performance and flexible thermoelectric films by screen printing solution-processed nanoplate crystals. *Sci. Rep.* **6**, 33135; doi: 10.1038/srep33135 (2016).

## Supplementary Material

Supplementary Information

## Figures and Tables

**Figure 1:**
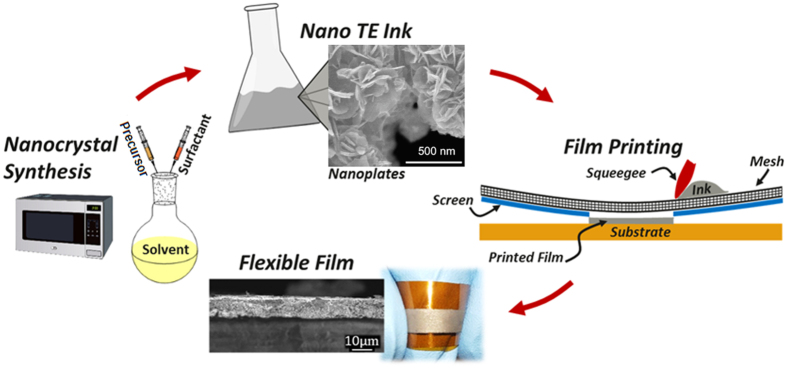
Schematic illustration of overall fabrication process for the flexible thermoelectric films, including nanocrystal synthesis, nano-ink processing, screen printing of thermoelectric films on flexible substrate, and sintered flexible films.

**Table 1:** The thermoelectric performance comparison between our work and previous reported n-type flexible thermoelectric films.

Materials details	Power factor (mWm^−1^K^−2^)	Peak/room T^*^ ZT	Ref.	Fabrication methods
Bi_2_Te_2.8_Se_0.2_	0.56	0.43	(Ours)	Screen printing
Bi_2_Te_3_	1.33	0.35^*^	16	Screen printing
Bi_2_Te_3_ + Epoxy	0.28	0.31^*^	15	Dispenser printing
CNT	0.15	N.A.	23	Drop casting
WS_2_	0.007	N.A.	24	Vacuum filtration
TiS_2_-Polymer	0.45	0.28	25	Electrochemical intercalation
CNT-PEDOT-TDAE	1.05	~0.5^*^	26	Spraying and spin coating
