# Supplementary material for: High-performance and flexible thermoelectric films by screen printing solution-processed nanoplate crystals
Source: Sci Rep. 2016 Sep 12;6:33135. doi: 10.1038/srep33135 (PMC5018881; doi:10.1038/srep33135)
Supplement: Supplementary Information [file srep33135-s1.pdf]

High-performance and flexible thermoelectric devices by screen printing solution-processed nanoplate crystals

Tony Varghese,[1] Courtney Hollar,[2] Joseph Richardson,[3] Nicholas Kempf,[2] Chao Han,[2] Pasindu Gamarachchi,[2] David Estrada,[1] Rutvik J. Mehta,[4] Yanliang Zhang[1,2*]

[1]*Department of Materials Science and Engineering, Boise State University, Boise, ID 83725, United States*

[2]*Department of Mechanical and Biomedical Engineering, Boise State University, Boise, ID 83725, United States*

[3]*Department of Electrical and Computer Engineering, Boise State University, Boise, ID 83725, United States*

[4]*ThermoAura, inc. 132 B. Railroad Avenue, Colonie, NY 12205, United States*

*Email: yanliangzhang@boisestate.edu

Measurement of in-plane thermal conductivity of thermoelectric films.

The in-plane thermal conductivity of the printed films was measured using the parallel thermal conductance method.[1] Two separate measurements were conducted in order to determine the baseline thermal conductance of the test setup and total thermal conductance including the test setup and the sample, the difference of which yielded the sample thermal conductance. A freestanding film (15 mm × 4 mm × 0.1 mm) was prepared by removing the substrate after cold compaction and before sintering when the adhesion between the film and the substrate was still very weak. The film was then sintered at the same conditions as the films on substrate. The electrical conductivity and Seebeck coefficient of the 100 μm thick freestanding film and the 10 μm thick film shown in Figure 3 are within 6%, indicating nearly identical thermoelectric properties independent of the sample thicknesses.

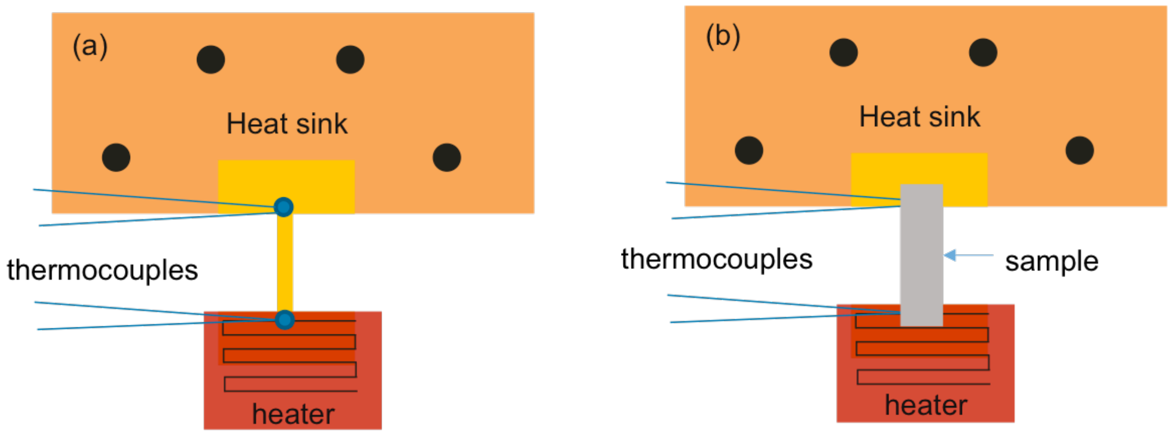

Figure S1. (a) Sample holder for measuring in-plane thermal conductivity, (b) A thermoelectric film sample mounted in the sample holder.

As shown in Figure S1, a sample holder was fabricated to secure the film sample during measurement. A Kapton strip was used as the sample holder due to its low thermal conductivity, which minimizes the baseline thermal conductance. A strainguage heater was attached to the hot side of the sample holder to provide heat sources while the cold side was mounted to a copper heat sink. The measurement was

conducted in a vacuum cryostat chamber at 10^{-5} Torr to minimize convection losses. In addition, a radiation shield was surrounding the test setup to minimize radiation losses.

The first baseline measurement determines the baseline thermal conductance $C_{baseline}$ of the sample holder using equation [1].

$$P = I^2 R = C_{baseline} \Delta T \qquad [1]$$

The second measurement determines the total thermal conductance C_{total} of the sample and the sample holder.

$$P = I^2 R = C_{total} \Delta T \qquad [2]$$

The thermal conductivity of the sample is determined using the following equation.

$$K = (C_{total} - C_{baseline}) \cdot L/A \qquad [3]$$

To calibrate the test setup, the thermal conductivity of a 0.2 mm thick glass slide was measured, which was within 5% of the reference value.

1. Zawilski, B.M., Littleton R.T., and Tritt T.M., Description of the parallel thermal conductance technique for the measurement of the thermal conductivity of small diameter samples. *Review of Scientific Instruments*, **72** (3),1770-1774 (2001).
